# Toll-like receptor 2 contributes to antibacterial defence against pneumolysin-deficient pneumococci

**DOI:** 10.1111/j.1462-5822.2007.01035.x

**Published:** 2008-01

**Authors:** Mark C Dessing, Sandrine Florquin, James C Paton, Tom van der Poll

**Affiliations:** 1 Center of Infection and Immunity Amsterdam (CINIMA) the Netherlands; 2 Center of Experimental and Molecular Medicine the Netherlands; 3 Department of Pathology, Academic Medical Center, University of Amsterdam the Netherlands; 4 School of Molecular and Biomedical Science, University of Adelaide Adelaide, Australia

## Abstract

Toll-like receptors (TLRs) are pattern recognition receptors that recognize conserved molecular patterns expressed by pathogens. Pneumolysin, an intracellular toxin found in all *Streptococcus pneumoniae* clinical isolates, is an important virulence factor of the pneumococcus that is recognized by TLR4. Although TLR2 is considered the most important receptor for Gram-positive bacteria, our laboratory previously could not demonstrate a decisive role for TLR2 in host defence against pneumonia caused by a serotype 3 *S. pneumoniae*. Here we tested the hypothesis that in the absence of TLR2, *S. pneumoniae* can still be sensed by the immune system through an interaction between pneumolysin and TLR4. C57BL/6 wild-type (WT) and TLR2 knockout (KO) mice were intranasally infected with either WT *S. pneumoniae* D39 (serotype 2) or the isogenic pneumolysin-deficient *S. pneumoniae* strain D39 PLN. TLR2 did not contribute to antibacterial defence against WT *S. pneumoniae* D39. In contrast, pneumolysin-deficient *S. pneumoniae* only grew in lungs of TLR2 KO mice. TLR2 KO mice displayed a strongly reduced early inflammatory response in their lungs during pneumonia caused by both pneumolysin-producing and pneumolysin-deficient pneumococci. These data suggest that pneumolysin-induced TLR4 signalling can compensate for TLR2 deficiency during respiratory tract infection with *S. pneumoniae.*

## Introduction

*Streptococcus pneumoniae* is the most common cause of community-acquired pneumonia (Bernstein, 1999; Campbell, 1999). Infections caused by *S. pneumoniae* are increasingly difficult to treat due to the emergence of antibiotic-resistant strains (Pikis *et al*., 1995; Schreiber and Jacobs, 1995). Increased knowledge of the first interaction between *S. pneumoniae* and host immune cells may facilitate the development of novel prophylactic and therapeutic tools to combat pneumococcal infections. In this respect, Toll-like receptors (TLRs), a family of pattern recognition receptors that are capable of recognizing conserved molecular patterns expressed by pathogens, are of particular interest (Kawai and Akira, 2005; Paterson and Mitchell, 2006).

The pneumococcal cell wall consists of several proteins and enzymes that contribute to the virulence of the pathogen and the pathogenesis of pneumonia (Jedrzejas, 2001). Pneumolysin is an intracellular toxin found in *S. pneumoniae*, which is produced by all clinical isolates and is an important factor for the virulence of the pneumococcus (Hirst *et al*., 2004). Indeed, mice infected with a pneumolysin-deficient strain of *S. pneumoniae* showed a reduced lethality and a diminished inflammatory response compared with mice infected with a normal, pneumolysin-producing strain (Berry *et al*., 1989; Benton *et al*., 1995; Canvin *et al*., 1995; Rubins *et al*., 1995; Berry and Paton, 2000; Kadioglu *et al*., 2002). At sublytic dose, pneumolysin affects polymorphonuclear cell activity, including respiratory burst, degranulation, chemotaxis and bactericidal activity (Paton and Ferrante, 1983). Furthermore, pneumolysin activates the classical pathway of complement and induces cytokine production by macrophages and monocytes (Houldsworth *et al*., 1994; Paton, 1996; Cockeran *et al*., 2002). At lytic dose, pneumolysin forms ring-shaped pores in cholesterol-containing cell membranes, which results in cell death (Alouf, 2000; Gilbert, 2002). Recent work has suggested that the immune system recognizes pneumolysin through TLR4 (Malley *et al*., 2003; Srivastava *et al*., 2005). Both pneumolysin-induced cytokine production and pneumolysin-induced apoptosis are mediated through TLR4 (Malley *et al*., 2003; Srivastava *et al*., 2005). In a model of nasopharyngeal colonization by *S. pneumoniae*, the interaction between pneumolysin and TLR4 was found to be essential for preventing invasive disease (Malley *et al*., 2003). Our laboratory reported a protective role of TLR4 during infection of the lower respiratory tract by *S. pneumoniae*, demonstrating an enhanced growth of bacteria in lungs of TLR4-deficient mice (Branger *et al*., 2004).

Within the family of TLRs, TLR2 has been implicated as the major pattern recognition receptor for ligands derived from Gram-positive bacteria (Yoshimura *et al*., 1999; Han *et al*., 2003; Schroder *et al*., 2003; Kawai and Akira, 2005). However, our laboratory recently demonstrated that TLR2 does not play a key role in host resistance to pneumonia caused by a serotype 3 strain of *S. pneumoniae* (Knapp *et al*., 2004). We here hypothesized that TLR2 knockout (KO) mice have an intact protective immune response against *S. pneumoniae*, because they are still capable of activating their immune system through an interaction between pneumolysin and TLR4. If this assumption is true, TLR2 KO mice would display a reduced antibacterial defence against pneumolysin-deficient *S. pneumoniae*, considering that these modified bacteria, devoid of a major TLR4 ligand, would primarily express TLR2 ligands. Therefore, in the present study we compared the response of TLR2 KO and wild-type (WT) mice during respiratory tract infection with WT and pneumolysin-deficient *S. pneumoniae*.

## Results

### Toll-like receptor 2 does not contribute to host defence and pulmonary inflammation against pneumonia caused by WT *S. pneumoniae* D39

**Fig. 1:**
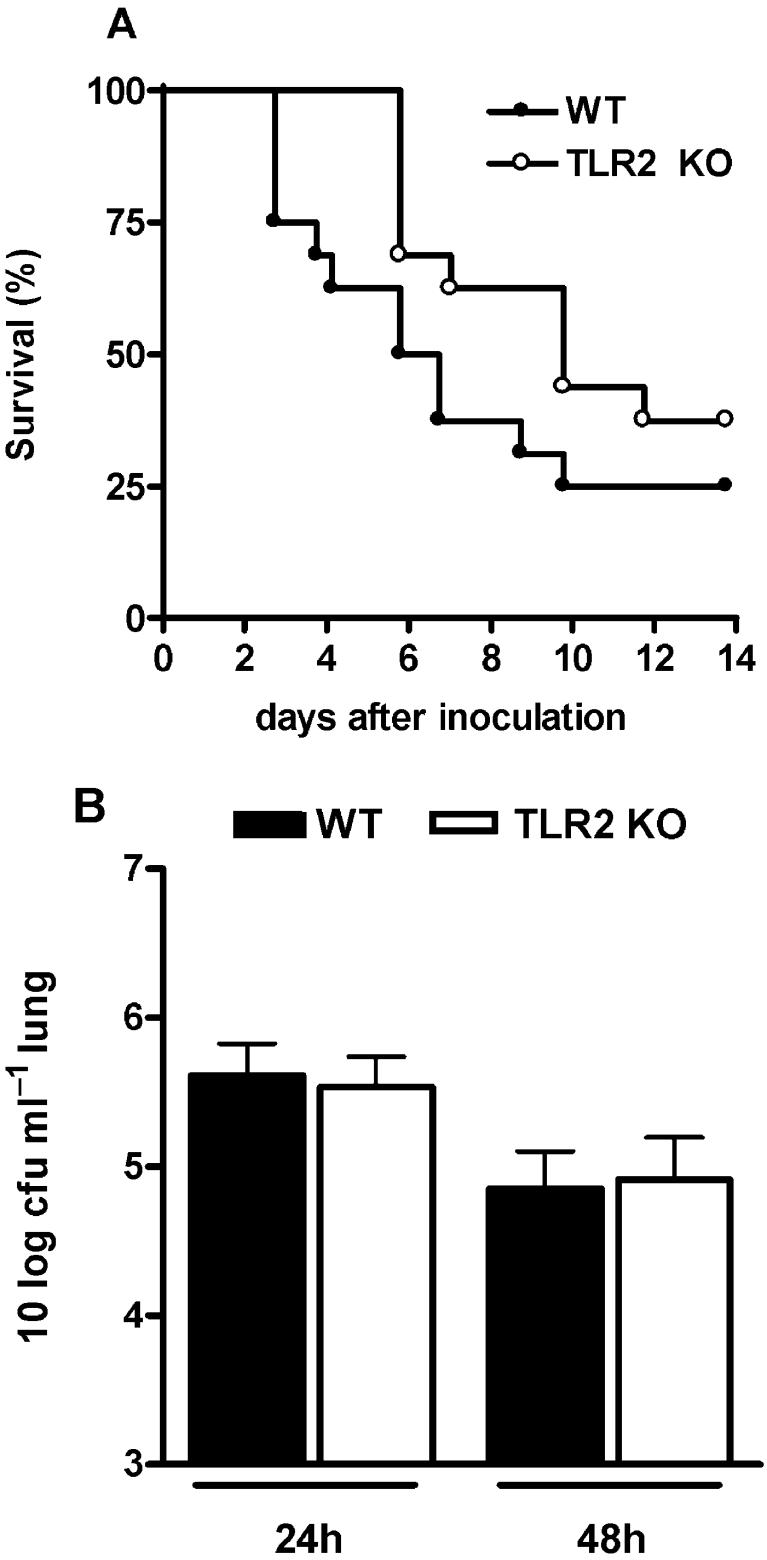
TLR2 does not contribute to host defence against WT *S. pneumoniae*. Survival (A) and bacterial outgrowth (B) of WT mice (closed symbols or bars) and TLR2 KO mice (open symbols or bars) with 5 × 10^7^ cfu *S. pneumoniae* D39. Mortality was assessed four times daily for 14 days (*n* = 16 per group). Bacterial loads in WT mice and TLR2 KO mice were determined 24 and 48 h after infection. Data of bacterial loads are mean ± SEM (*n* = 7–8 per group at each time point).

We previously showed that TLR2 KO mice are indistinguishable from WT mice with regard to bacterial outgrowth and mortality after intranasal infection with a serotype 3 *S. pneumoniae* strain (Knapp *et al*., 2004). Considering that the pneumolysin-deficient strain used here is a serotype 2 (derived from *S. pneumoniae* D39), we first investigated the impact of TLR2 deficiency on the course of pneumonia caused by WT *S. pneumoniae* D39 (Fig. 1). Mortality did not differ between TLR2 KO and WT mice after intranasal infection with *S. pneumoniae* D39; if anything, TLR2 KO mice displayed a slightly reduced mortality (62.5%) although the difference with WT mice (75% mortality) was not significant (*P* = 0.13; Fig. 1A). We next determined bacterial loads in whole lung homogenates at 24 and 48 h after infection, i.e. at time points just before the first mice started dying (Fig. 1B). At both 24 and 48 h, bacterial loads were identical in lungs of TLR2 KO and WT mice. Together these data extend our earlier study using a serotype 3 *S. pneumoniae* strain (Knapp *et al*., 2004), showing that TLR2 does not contribute to a protective immune response during pneumonia caused by a serotype 2 pneumococcus.

### Toll-like receptor 2 (TLR2) deficiency modestly attenuates the inflammatory response induced by WT *S. pneumoniae* D39

Cytokines and chemokines play an important role in the antibacterial defence against bacterial pneumonia (Moore and Standiford, 2001; Knapp *et al*., 2005). We therefore determined the concentrations of tumour necrosis factor (TNF)-α, interleukin (IL)-1β, IL-10, macrophage inflammatory protein (MIP)-2 and cytokine-induced neutrophil chemoattractant (KC) in whole lung homogenates obtained 24 and 48 h after inoculation (Table 1). Although in general the pulmonary concentrations of these mediators were lower in TLR2 KO mice, the differences with WT mice were statistically significant only for KC (*P* < 0.005 at 24 and 48 h post infection) and IL-1β (*P* < 0.05 at 48 h). To further investigate lung inflammation, we determined pulmonary myeloperoxidase (MPO) levels, reflecting the whole-organ neutrophil content, in TLR2 KO mice and WT mice (Table 1). Similar to cytokine and chemokine levels, MPO concentrations were modestly lower in TLR2 KO, significantly so at 48 h post infection (*P* < 0.01). Moreover, total lung inflammation scores, determined from lung tissue slides prepared 24 and 48 h after infection with *S. pneumoniae* D39, were similar in WT and TLR2 KO mice (Table 1). Together, these data obtained with a serotype 2 pneumococcus confirm our earlier data generated with a serotype 3 *S. pneumoniae* (Knapp *et al*., 2004), establishing that TLR2 plays a modest role in the induction of a pulmonary inflammatory response to respiratory tract infection with WT *S. pneumoniae*.

**Table 1:** Parameters of lung inflammation in TLR2 KO and WT mice 24 and 48 h after infection with WT *S. pneumoniae* D39.

	24 h		48 h	
		
	WT	TLR2 KO	WT	TLR2 KO
TNF-α	1229 ± 351	1026 ± 212	500 ± 92	361 ± 63
IL-1β	4029 ± 599	3248 ± 495	2874 ± 594	1462 ± 329*
IL-10	62 ± 23	48 ± 14	123 ± 51	72 ± 13
MIP-2	5912 ± 876	5689 ± 1039	1447 ± 177	1182 ± 243
KC	5943 ± 867	2244 ± 510**	3481 ± 339	695 ± 90**
MPO	7668 ± 1123	6158 ± 2317	9183 ± 2365	4057 ± 578***
TLIS	16.7 ± 0.9	16.8 ± 1.0	13.5 ± 0.6	15.5 ± 0.8

Mice were intranasally infected with 5 × 10^7^ cfu WT *S. pneumoniae* D39; whole lung homogenates were obtained 24 and 48 h thereafter. Data are means ± SEM (*n* = 6–8 per group).

* *P* < 0.05 versus WT mice.

** *P* < 0.005 versus WT mice.

*** *P* < 0.01 versus WT mice.

TNF-α, IL-1β, IL-10, MIP-2 and KC values are in pg ml^−1^; MPO values are in ng ml^−1^; TLIS (total lung inflammation score) in arbitrary units.

### Toll-like receptor 2 limits the outgrowth of pneumolysin-deficient *S. pneumoniae* PLN

**Fig. 2:**
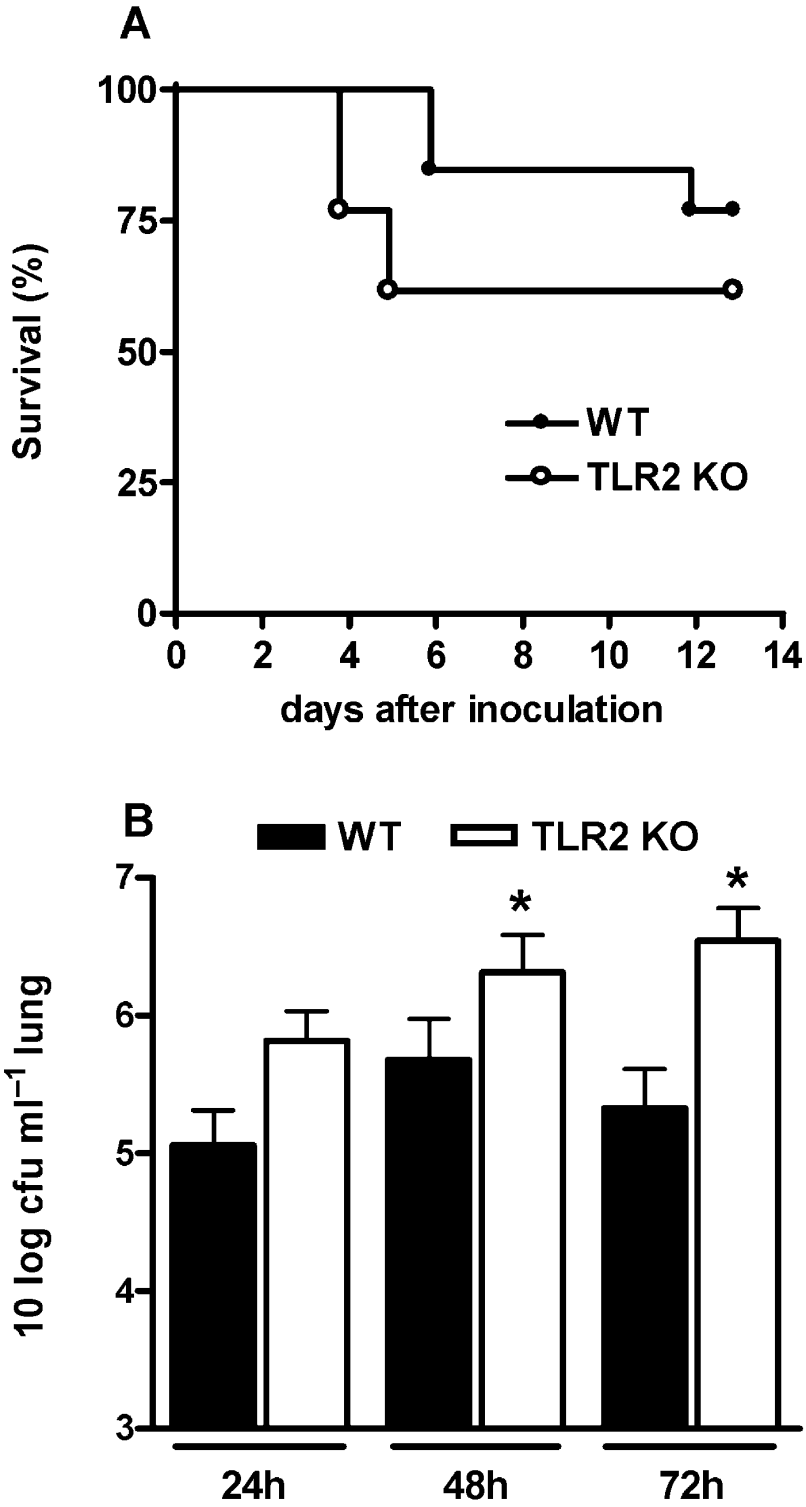
TLR2 limits outgrowth of pneumolysin-deficient *S. pneumoniae* PLN. Survival (A) and bacterial outgrowth (B) of WT mice (closed symbols or bars) and TLR2 KO mice (open symbols or bars) with 5 × 10^7^ cfu *S. pneumoniae* PLN. Mortality was assessed four times daily for 14 days (*n* = 13 per group). Bacterial loads in WT mice and TLR2 KO mice were determined 24, 48 and 72 h after infection. Data of bacterial loads are mean ± SEM (*n* = 7–8 per group at each time point) *P < 0.05 versus WT mice.

Having established that TLR2 is not essential for host defence against WT *S. pneumoniae* D39, we next infected TLR2 KO and WT mice with the isogenic mutant *S. pneumoniae* PLN (Fig. 2). As expected (Berry *et al*., 1989), *S. pneumoniae* PLN was less virulent, in particular in WT mice. Only 23% of WT mice died during a 2 week follow-up, versus 38% of TLR2 KO mice (not significant for the difference between mouse strains; *P* = 0.29; Fig. 2A). Interestingly, TLR2 KO mice started to die after 3 days, whereas the first deaths among WT mice occurred after 5 days. To obtain insight in the growth of *S. pneumoniae* PLN during the infection (i.e. before the first mice started dying), we infected TLR2 KO and WT mice with *S. pneumoniae* PLN and determined bacterial loads in whole lung homogenates at 24, 48 and 72 h thereafter (Fig. 2B). Whereas the bacterial burdens were not significantly different between TLR2 KO and WT mice 24 h post infection, at 48 and 72 h TLR2 KO displayed significantly higher bacterial loads in their lungs than WT mice (both *P* < 0.05). Remarkably, whereas *S. pneumoniae* PLN did not further grow in the lungs of WT mice from 24 h after infection onward, which is in line with a previous investigation (Canvin *et al*., 1995), the bacterial load increased more than 10-fold in lungs of TLR2 KO mice between 24 and 72 h after inoculation. Hence, these data show that TLR2 serves to limit the growth of *S. pneumoniae* PLN during pneumonia.

### Toll-like receptor 2 (TLR2) deficiency reduces lung inflammation induced by *S. pneumoniae* PLN

**Fig. 3:**
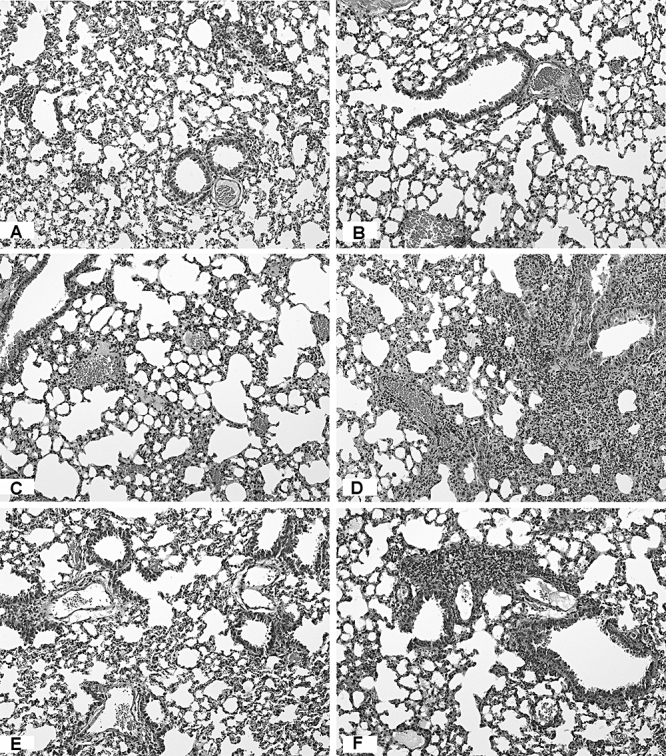
Lung histology in WT and TLR2 KO mice after infection with *S. pneumoniae* PLN. Representative lung tissue slides from WT mice (A, C and E) and TLR2 KO mice (B, D and F) after infection with 5 × 10^7^ cfu *S. pneumoniae* PLN. Mice were sacrificed after 24 h (A and B), 48 h (C and D) or 72 h (E and F). HE staining: magnification 4×.

To further investigate the role of TLR2 during infection with *S. pneumoniae* PLN, we determined TNF-α, IL-1β, IL-10, MIP-2, KC and MPO levels in whole lung homogenates obtained 24, 48 and 72 h after inoculation (Table 2). At 24 h post infection, pulmonary cytokine and chemokine levels were lower in TLR2 KO mice, significantly so for IL-1β (*P* < 0.05). In addition, lung MPO levels were lower in TLR2 KO mice at this time point (*P* < 0.05). In contrast, at 48 and 72 h after infection, when TLR2 KO mice displayed higher bacterial burdens in their lungs, the pulmonary concentrations of cytokines, chemokines and MPO did not differ between TLR2 KO and WT mice. Histopathological analyses of lung tissue slides demonstrated reduced lung inflammation in TLR2 KO mice at 24 h, but increased lung inflammation at 48 and 72 h (Table 2). Representative lung tissue slides from WT and TLR2 KO mice 24, 48 and 72 h after infection with *S. pneumoniae* PLN are shown in Fig. 3.

**Table 2:** Parameters of lung inflammation in TLR2 KO and WT mice 24, 48 and 72 h after infection with pneumolysin-deficient *S. pneumoniae* PLN.

	24 h		48 h		72 h	
			
	WT	TLR2 KO	WT	TLR2 KO	WT	TLR2 KO
TNF-α	325 ± 77	295 ± 87	195 ± 34	195 ± 34	506 ± 114	522 ± 163
IL-1β	2414 ± 519	903 ± 496*	552 ± 215	802 ± 200	1724 ± 638	2092 ± 956
IL-10	BD	BD	BD	BD	135 ± 27	153 ± 63
MIP-2	2703 ± 1033	1956 ± 1001	1919 ± 194	2720 ± 649	1029 ± 589	1297 ± 692
KC	1781 ± 791	600 ± 158	1700 ± 980	610 ± 188	1385 ± 323	958 ± 391
MPO	3173 ± 391	1666 ± 411*	1675 ± 424	2205 ± 226	2722 ± 251	2402 ± 500
TLIS	7.7 ± 2.0	3.1 ± 1.2	6.9 ± 1.6	11.9 ± 2.1	5.6 ± 0.9	8.7 ± 0.8*

Mice were intranasally infected with 5 × 10^7^ cfu *S. pneumoniae* PLN; whole lung homogenates were obtained 24, 48 and 72 h thereafter. Data are means ± SEM (*n* = 6–8 per group).

* *P* < 0.05 versus WT mice.

TNF-α, IL-1β, IL-10, MIP-2 and KC values are in pg ml^−1^; MPO values are in ng ml^−1^; TLIS (total lung inflammation score) in arbitrary units.

BD, below detection limit.

### Toll-like receptor 2 (TLR2) deficiency strongly impairs the early inflammatory response to both *S. pneumoniae* D39 and *S. pneumoniae* PLN

**Fig. 4:**
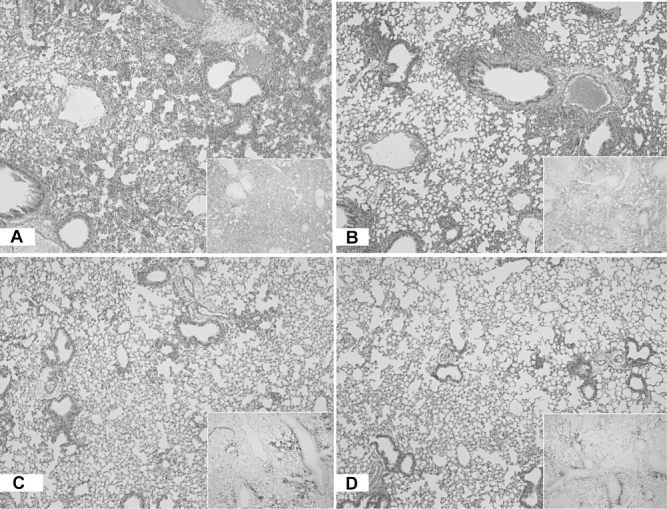
Reduced lung inflammation in TLR2 KO mice early after infection with *S. pneumoniae* D39 or *S. pneumoniae* PLN. Representative lung tissue slides from WT mice (A and C) and TLR2 KO mice (B and D) 6 h after infection with 5 × 10^7^ cfu *S. pneumoniae* D39 (A and B) or *S. pneumoniae* PLN (C and D). HE staining: magnification 4×. Insets show Ly-6G staining.

The role of TLR2 in lung inflammation later in the course of pneumonia could have been obscured by the growing bacterial load in TLR2 KO mice (that is, at 48 and 72 h after infection, TLR2 deficiency could be compensated for by the higher bacterial load, providing a more potent proinflammatory stimulus via TLR2-independent pathways). An earlier study performed at our laboratory has shown an important role for TLR2 in the early host defence against *S. pneumoniae* pneumonia using serotype 3 (Knapp *et al*., 2004). TLR2 could also be more important for the early host inflammatory response to *S. pneumoniae* with serotype 2. Thus, we intranasally inoculated TLR2 KO and WT mice with *S. pneumoniae* D39 or *S. pneumoniae* PLN and evaluated their response to the infection 6 h later (Table 3). The bacterial loads in lungs of TLR2 KO mice and WT mice were similar at this early time point during infection with *S. pneumoniae* D39 or *S. pneumoniae* PLN. Interestingly, compared with WT mice, TLR2 KO mice showed a strongly reduced capacity to respond to both *S. pneumoniae* D39 and *S. pneumoniae* PLN; the lung concentrations of TNF-α, IL-1β, MIP-2 and KC were much lower in TLR2 KO mice (*P* < 0.05 to *P* < 0.001) (Table 3). In addition, histopathological analyses of lung tissue slides demonstrated a significantly reduced inflammation in lungs of TLR2 KO mice 6 h after infection with *S. pneumoniae* D39 or *S. pneumoniae* PLN (Fig. 4). Of note, some inflammatory responses to *S. pneumoniae* PLN were more strongly reduced in TLR2 KO mice than the inflammatory responses to *S. pneumoniae* D39. For KC, MIP-2 and TNF-α, there was a significant interaction (*P* < 0.001, *P* = 0.034 and *P* = 0.007, respectively); the effect of the pneumococcal strain used depended on the mouse strain. In particular, whereas in WT mice *S. pneumoniae* D39 and PLN induced a similar early TNF-α response in the lungs, the pulmonary levels of this crucially protective cytokine in the early response to pneumococcal pneumonia (van der Poll *et al*., 1997; Rijneveld *et al*., 2001) were more than 4-fold lower in TLR2 KO mice after infection with *S. pneumoniae* PLN versus 2-fold lower after inoculation with *S. pneumoniae* D39. In addition, whereas overall *S. pneumoniae* PLN elicited less profound histopathological alterations in lung tissue than *S. pneumoniae* D39, the difference in total lung histology scores between TLR2 KO and WT mice was especially clear after infection with the pneumolysin-deficient strain.

**Table 3:** Role of TLR2 in the early inflammatory response in the lungs after infection with *S. pneumoniae* D39 or PLN.

	D39		PLN	
		
	WT	TLR2 KO	WT	TLR2 KO
cfu	6.5 ± 1.4 × 10^6^	5.5 ± 1.3 × 10^6^	13.2 ± 1.3 × 10^6^	10.6 ± 1.8 × 10^6^
TNF-α	9 781 ± 780	4 656 ± 375**	9 124 ± 1 203	2270 ± 236**
IL-1β	5 754 ± 714	3 566 ± 348*	18 963 ± 2 522	8716 ± 1939*
IL-10	31 ± 2	32 ± 3	BD	BD
MIP-2	42 193 ± 2 529	23 855 ± 3 396***	9 114 ± 1 112	2643 ± 505**
KC	61 120 ± 2 879	23 266 ± 3 124***	16 776 ± 2 314	4783 ± 1352**
MPO	76.7 ± 9.3	41.6 ± 3.4*	15.5 ± 3.4	12.4 ± 4.0
TLIS	18.4 ± 0.5	15.1 ± 0.8*	9.5 ± 0.9	5.3 ± 1.3*

Mice were intranasally infected with 5 × 10^7^ cfu *S. pneumoniae* D39 or PLN, and whole lung homogenates were obtained 6 h later. Data are means ± SEM (*n* = 8 per group).

* *P* < 0.05 versus WT mice.

** *P* < 0.001 versus WT mice.

*** *P* < 0.01 versus WT mice.

TNF-α, IL-1β, IL-10, MIP-2 and KC values are in pg ml^−1^; cfu values are in cfu ml^−1^ lung; MPO levels are in μg ml^−1^; TLIS (total lung inflammation score) in arbitrary units.

BD, below detection limit.

## Discussion

Pneumolysin is an essential virulence factor of *S. pneumoniae* (Hirst *et al*., 2004). Recent studies have identified TLR4 as a recognition receptor for pneumolysin in the respiratory tract (Malley *et al*., 2003; Srivastava *et al*., 2005). The interaction between pneumolysin and TLR4 was found to contribute to a protective immune response to *S. pneumoniae*, in particular in a model of upper airway colonization (Malley *et al*., 2003; Srivastava *et al*., 2005) and to a lesser extent during experimental lower respiratory tract infection (Branger *et al*., 2004). Although the pneumococcus expresses several potent TLR2 ligands (Yoshimura *et al*., 1999; Han *et al*., 2003; Schroder *et al*., 2003), our laboratory previously could not demonstrate a decisive role for TLR2 in host defence against pneumococcal pneumonia (Knapp *et al*., 2004). We here hypothesized that in the absence of TLR2, *S. pneumoniae* could still be sensed by the immune system through an interaction between pneumolysin and TLR4. The experiments described herein support this hypothesis: whereas the growth of WT pneumococci occurred to a similar extent in TLR2 KO and WT mice, the pneumolysin-deficient *S. pneumoniae* PLN strain only grew out in TLR2 KO mice. These data suggest that pneumolysin-induced TLR4 signalling can compensate for TLR2 deficiency during respiratory tract infection with *S. pneumoniae*.

In a series of elegant experiments Malley and coworkers demonstrated that pneumolysin is a ligand for TLR4 (Malley *et al*., 2003; Srivastava *et al*., 2005). Purified pneumolysin was shown to activate cells via a TLR4-dependent, TLR2-independent pathway, accomplished by a physical interaction between pneumolysin and TLR4 (Malley *et al*., 2003; Srivastava *et al*., 2005). Interestingly, pneumolysin induced proinflammatory responses in primary macrophages in synergy with TLR2 ligands derived from *S. pneumoniae*, in particular peptidoglycan and whole pneumococcal cell walls (Malley *et al*., 2003; Srivastava *et al*., 2005), suggesting that during infection with intact pneumococci, the combined action of TLR4 and TLR2 may facilitate an optimal innate immune response. Such roles for these two distinct TLRs is further corroborated by findings that, in the human embryonic kidney cell line 293, transfection of either TLR2 or TLR4 conferred responsiveness to *S. pneumoniae* (Koedel *et al*., 2003). Thus far, the isolated roles of either TLR4 or TLR2 in host defence against *S. pneumoniae in vivo* have been investigated in a number of studies. The most dramatic phenotype was reported in the original publication by Malley *et al*. (Malley *et al*., 2003; Srivastava *et al*., 2005), showing that C3H/HeJ mice, which carry a loss-of-function *tlr4* mutation, are more susceptible to pneumococcal colonization after nasopharyngeal challenge, eventually resulting in invasive infection, bacteremia and death. Our laboratory found a more modest protective role for TLR4 during lower respiratory tract infection by *S. pneumoniae*, as reflected by a reduced survival and a slightly enhanced bacterial outgrowth after intranasal infection of C3H/HeJ mice with a relatively low infectious dose (Branger *et al*., 2004). TLR2 KO mice demonstrated an increased disease severity together with a moderately enhanced bacterial growth in the central nervous system during meningitis induced by intracisternal injection of pneumococci (Echchannaoui *et al*., 2002; Koedel *et al*., 2003). In contrast, our group could not demonstrate a protective role for TLR2 in pneumonia caused by *S. pneumoniae*, showing similar bacterial multiplication and lethality after intranasal infection of TLR2 KO and WT mice (Knapp *et al*., 2004). A limited role for TLR2 during infection with WT *S. pneumoniae* is further supported by a recent study in which intact pneumococci were administered intraperitoneally (Khan *et al*., 2005), although TLR2 KO mice displayed a modestly slower clearance of *S. pneumoniae* from their nasopharynx in another investigation (van Rossum *et al*., 2005). Altogether these studies suggest that TLR2 at best plays a modest role in host defence against *S. pneumoniae* airway infection, and led us to hypothesize that intact TLR4 signalling through pneumolysin may balance the lack of TLR2 signalling. We tested this hypothesis by infecting TLR2 KO mice with pneumolysin-deficient *S. pneumoniae*, arguing that these bacteria, devoid of a major TLR4 ligand, predominantly express TLR2 ligands. Indeed, whereas antibacterial defence in TLR2 KO mice was unimpaired during infection with *S. pneumoniae* D39, infection with *S. pneumoniae* PLN resulted in enhanced outgrowth in these mice. If our hypothesis is correct, inoculation of WT *S. pneumoniae* D39 in TLR2 × 4 double KO mice should result in a comparable setting as pneumolysin-deficient *S. pneumoniae* in TLR2 KO mice, i.e. absence of TLR2 *and* TLR4 signalling. Our first preliminary results show that indeed this is the case: growth of WT *S. pneumoniae* D39 was significantly higher in the lungs of TLR2 × 4 double KO mice compared with WT mice 48 h after inoculation (data not shown). In line, Albiger *et al*. (2005) recently showed that mice deficient of the TLR2- and TLR4-common intracellular adaptor molecule MyD88 also displayed an enhanced bacterial outgrowth in MyD88 KO mice compared with WT mice.

The early inflammatory response is an essential component of host defence in this model of pneumococcal pneumonia, as documented by previous studies in which the early cytokine response was inhibited (van der Poll *et al*., 1997; Rijneveld *et al*., 2001). Although TLR2 KO mice displayed a reduced inflammatory response 6 h after infection with either *S. pneumoniae* D39 or PLN, some responses were more strongly diminished after infection with the pneumolysin-deficient strain. This was in particular true for the early TNF-α response. Considering that especially low TNF-α concentrations in the lungs early after induction of pneumococcal pneumonia are important for limiting the growth of *S. pneumoniae* (van der Poll *et al*., 1997; Rijneveld *et al*., 2001), this differential response may have contributed to the enhanced growth of *S. pneumoniae* PLN in TLR2 KO mice. In addition, mediators other than measured in this study could contribute to this finding. Of note, TLR2 KO mice still display an induction of cytokines and chemokines when infected with pneumolysin-deficient *S. pneumoniae*, suggesting that other pattern recognition receptors contribute to this response, In this respect, the recent finding that TLR9 can recognize pneumococcall DNA is of relevance (Mogensen *et al*., 2006). Moreover, although histopathological analysis of lung tissue showed diminished lung inflammation in TLR2 KO mice during the early course of infection with *S. pneumoniae* PLN, which is in line with a TLR2-dependent immune response, during the later phase of pneumonia lung inflammation of TLR2 KO mice was enhanced compared with WT mice, which corresponded with the higher bacterial loads. This finding suggests that, in the presence of a high bacterial burden, *S. pneumoniae* PLN is able to elicit significant lung inflammation via a TLR2-independent route. Hence, it is conceivable that the early recognition of *S. pneumoniae* and, thereby, the initial inflammatory response in the airways at least in part are mediated by TLR2, through an interaction between this receptor and the various TLR2 ligands expressed by the pneumococcus (Yoshimura *et al*., 1999; Han *et al*., 2003; Schroder *et al*., 2003), but that, during a more established infection with a higher bacterial burden, the TLR2-induced inflammation is ‘overruled’ by other pathways stimulated by TLR2-independent pneumococcal antigens.

Our results exemplify the complex interactions at play during the first encounter between the host, expressing multiple pattern recognition receptors, and an intact pathogen, expressing multiple virulence factors and pathogen-associated molecular patterns. Whereas during infection of TLR2 KO mice with WT pneumococci, the interaction between TLR4 and pneumolysin apparently is sufficient to maintain an adequate immune response, during infection of TLR2 KO mice with pneumolysin-deficient *S. pneumoniae*, the absence of the interaction between pneumococcal TLR2 ligands, such as lipoteichoic acid and peptidoglycan, cannot be compensated for by the TLR4 pneumolysin-mediated immune response. As such, our data demonstrate redundancy at both the microbial site and the site of the host during airway infection by *S. pneumoniae*.

## Experimental procedures

### Animals

C57BL/6 WT mice were purchased from Charles Rivers (Maastricht, the Netherlands). TLR2 KO mice (Takeuchi *et al*., 1999), backcrossed to a C57BL/6 genetic background six times, were bred in the animal facility of the Academic Medical Center in Amsterdam. Sex- and age-matched (10–12 weeks) mice were used in all experiments. All experiments were approved by the Animal Care and Use Committee of the University of Amsterdam.

### Design

The experimental procedures to induce pneumonia have been described earlier (Branger *et al*., 2004; Knapp *et al*., 2004; Dessing *et al*., 2007). *S. pneumoniae* serotype 2 (strain D39) and isogenic pneumolysin-deficient *S. pneumoniae* (strain PLN) (Berry *et al*., 1989) were grown for 5 h to mid-logarithmic phase at 37°C using Todd–Hewitt broth (Difco, Detroit, MI), harvested by centrifugation at 1500 *g* for 15 min, and washed twice in sterile isotonic saline. A total of 50 μl containing 5 × 10^7^ colony-forming units (cfu) were inoculated intranasally in mice which were lightly anesthetized by inhalation of isoflurane (Upjohn, Ede, the Netherlands). Mice were killed 6, 24 or 48 h after infection with *S. pneumoniae* D39, or 6, 24, 48 or 72 h after infection with *S. pneumoniae* PLN. In separate studies, survival of mice was determined during a 2 week follow-up.

### Measurement of bacterial loads

Lung bacterial loads were determined as described earlier (Branger *et al*., 2004; Knapp *et al*., 2004; Dessing *et al*., 2007). Briefly, mice were sacrificed, and blood and lungs were collected. Lungs were homogenized at 4°C in 5 vols of sterile isotonic saline with a tissue homogenizer (Biospect Products, Bartlesville, OK). Serial 10-fold dilutions in sterile isotonic saline were made from these homogenates (and blood), and 50 μl vols were plated onto sheep-blood agar plates and incubated overnight at 37°C and 5% CO_2_.

### Histology

Lungs for histology were fixed in 10% formalin and embedded in paraffin. Four millimetre sections were stained with haematoxylin and eosin (HE) and analysed by a pathologist who was blinded for groups. To score lung inflammation and damage, the entire lung surface was analysed with respect to the following parameters: bronchitis, oedema, interstitial inflammation, intra-alveolar inflammation, pleuritis and endothelialitis. Each parameter was graded on a scale of 0–4, with 0 as ‘absent’ and 4 as ‘severe’. The total ‘lung inflammation score’ was expressed as the sum of the scores for each parameter, the maximum being 24. Granulocyte staining was performed as described earlier by Ly-6G staining (Dessing *et al*., 2007).

### Assays

Lung homogenates were prepared as described earlier (Knapp *et al*., 2004). MPO was measured by ELISA (HyCult, Uden, the Netherlands). TNF-α, IL-1β, IL-10, MIP-2 and KC were measured by ELISA (R and D Systems, Abingdon, UK).

### Statistical analysis

Statistics were performed with GraphPad Prism version 4.00 for Windows (GraphPad Software, San Diego, CA). All data are given as means ± SEM. Differences between groups were analysed using Mann–Whitney *U*-test. For survival analyses, Kaplan-Meier analysis followed by log rank test was performed. To analyse the effect of the used bacterial strains and their interactions in more detail in WT and TLR2 KO mice 6 h after inoculation, results of the groups were compared using an anova. Data were checked for normal distribution and equal variances using the residuals. All variables were normally distributed, but MIP-2, IL-1β and TNF-α were ln-transformed to obtain equal variances. Later statistics was performed using spss statistical software version 12.0.1 (SPSS, Chicago, IL). A value of *P* < 0.05 was considered statistically significant.

## References

[b1] Albiger B, Sandgren A, Katsuragi H, Meyer-Hoffert U, Beiter K, Wartha F (2005). Myeloid differentiation factor 88-dependent signalling controls bacterial growth during colonization and systemic pneumococcal disease in mice. Cell Microbiol.

[b2] Alouf JE (2000). Cholesterol-binding cytolytic protein toxins. Int J Med Microbiol.

[b3] Benton KA, Everson MP, Briles DE (1995). A pneumolysin-negative mutant of *Streptococcus pneumoniae* causes chronic bacteremia rather than acute sepsis in mice. Infect Immun.

[b4] Bernstein JM (1999). Treatment of community-acquired pneumonia – IDSA guidelines. Infectious Diseases Society of America. Chest.

[b5] Berry AM, Paton JC (2000). Additive attenuation of virulence of *Streptococcus pneumoniae* by mutation of the genes encoding pneumolysin and other putative pneumococcal virulence proteins. Infect Immun.

[b6] Berry AM, Yother J, Briles DE, Hansman D, Paton JC (1989). Reduced virulence of a defined pneumolysin-negative mutant of *Streptococcus pneumoniae*. Infect Immun.

[b7] Branger J, Knapp S, Weijer S, Leemans JC, Pater JM, Speelman P (2004). Role of Toll-like receptor 4 in gram-positive and gram-negative pneumonia in mice. Infect Immun.

[b8] Campbell GD (1999). Commentary on the 1993 American Thoracic Society guidelines for the treatment of community-acquired pneumonia. Chest.

[b9] Canvin JR, Marvin AP, Sivakumaran M, Paton JC, Boulnois GJ, Andrew PW, Mitchell TJ (1995). The role of pneumolysin and autolysin in the pathology of pneumonia and septicemia in mice infected with a type 2 pneumococcus. J Infect Dis.

[b10] Cockeran R, Durandt C, Feldman C, Mitchell TJ, Anderson R (2002). Pneumolysin activates the synthesis and release of interleukin-8 by human neutrophils in vitro. J Infect Dis.

[b11] Dessing MC, Knapp S, Florquin S, de Vos AF, van der Poll T (2007). CD14 facilitates invasive respiratory tract infection by *Streptococcus pneumoniae*. Am J Respir Crit Care Med.

[b12] Echchannaoui H, Frei K, Schnell C, Leib SL, Zimmerli W, Landmann R (2002). Toll-like receptor 2-deficient mice are highly susceptible to *Streptococcus pneumoniae* meningitis because of reduced bacterial clearing and enhanced inflammation. J Infect Dis.

[b13] Gilbert RJ (2002). Pore-forming toxins. Cell Mol Life Sci.

[b14] Han SH, Kim JH, Martin M, Michalek SM, Nahm MH (2003). Pneumococcal lipoteichoic acid (LTA) is not as potent as staphylococcal LTA in stimulating Toll-like receptor 2. Infect Immun.

[b15] Hirst RA, Kadioglu A, O'Callaghan C, Andrew PW (2004). The role of pneumolysin in pneumococcal pneumonia and meningitis. Clin Exp Immunol.

[b16] Houldsworth S, Andrew PW, Mitchell TJ (1994). Pneumolysin stimulates production of tumor necrosis factor alpha and interleukin-1 beta by human mononuclear phagocytes. Infect Immun.

[b17] Jedrzejas MJ (2001). Pneumococcal virulence factors: structure and function. Microbiol Mol Biol Rev.

[b18] Kadioglu A, Taylor S, Iannelli F, Pozzi G, Mitchell TJ, Andrew PW (2002). Upper and lower respiratory tract infection by *Streptococcus pneumoniae* is affected by pneumolysin deficiency and differences in capsule type. Infect Immun.

[b19] Kawai T, Akira S (2005). Pathogen recognition with Toll-like receptors. Curr Opin Immunol.

[b20] Khan AQ, Chen Q, Wu ZQ, Paton JC, Snapper CM (2005). Both innate immunity and type 1 humoral immunity to *Streptococcus pneumoniae* are mediated by MyD88 but differ in their relative levels of dependence on toll-like receptor 2. Infect Immun.

[b21] Knapp S, Wieland CW, van 't Veer C, Takeuchi O, Akira S (2004). Toll-like receptor 2 plays a role in the early inflammatory response to murine pneumococcal pneumonia but does not contribute to antibacterial defense. J Immunol.

[b22] Knapp S, Schultz MJ, Poll TV (2005). Pneumonia models and innate immunity to respiratory bacterial pathogens. Shock.

[b23] Koedel U, Angele B, Rupprecht T, Wagner H, Roggenkamp A, Pfister HW, Kirschning CJ (2003). Toll-like receptor 2 participates in mediation of immune response in experimental pneumococcal meningitis. J Immunol.

[b24] Malley R, Henneke P, Morse SC, Cieslewicz MJ, Lipsitch M, Thompson CM (2003). Recognition of pneumolysin by Toll-like receptor 4 confers resistance to pneumococcal infection. Proc Natl Acad Sci USA.

[b25] Mogensen TH, Paludan SR, Kilian M, Ostergaard L (2006). Live *Streptococcus pneumoniae*, *Haemophilus influenzae*, *and Neisseria meningitidis* activate the inflammatory response through Toll-like receptors 2,4, and 9 in species-specific patterns. J Leukoc Biol.

[b26] Moore TA, Standiford TJ (2001). Cytokine immunotherapy during bacterial pneumonia: from benchtop to bedside. Semin Respir Infect.

[b27] Paterson GK, Mitchell TJ (2006). Innate immunity and the pneumococcus. Microbiology.

[b28] Paton JC (1996). The contribution of pneumolysin to the pathogenicity of *Streptococcus pneumoniae*. Trends Microbiol.

[b29] Paton JC, Ferrante A (1983). Inhibition of human polymorphonuclear leukocyte respiratory burst, bactericidal activity, and migration by pneumolysin. Infect Immun.

[b30] Pikis A, Akram S, Donkersloot JA, Campos JM, Rodriguez WJ (1995). Penicillin-resistant pneumococci from pediatric patients in the Washington, DC, area. Arch Pediatr Adolesc Med.

[b31] van der Poll T, Keogh CV, Buurman WA, Lowry SF (1997). Passive immunization against tumor necrosis factor-alpha impairs host defense during pneumococcal pneumonia in mice. Am J Respir Crit Care Med.

[b32] Rijneveld AW, Florquin S, Branger J, Speelman P, Van Deventer SJ, van der Poll T (2001). TNF-alpha compensates for the impaired host defense of IL-1 type I receptor-deficient mice during pneumococcal pneumonia. J Immunol.

[b33] van Rossum AM, Lysenko ES, Weiser JN (2005). Host and bacterial factors contributing to the clearance of colonization by *Streptococcus pneumoniae* in a murine model. Infect Immun.

[b34] Rubins JB, Charboneau D, Paton JC, Mitchell TJ, Andrew PW, Janoff EN (1995). Dual function of pneumolysin in the early pathogenesis of murine pneumococcal pneumonia. J Clin Invest.

[b35] Schreiber JR, Jacobs MR (1995). Antibiotic-resistant pneumococci. Pediatr Clin North Am.

[b36] Schroder NW, Morath S, Alexander C, Hamann L, Hartung T, Zahringer U (2003). Lipoteichoic acid (LTA) of *Streptococcus pneumoniae* and *Staphylococcus aureus* activates immune cells via Toll-like receptor (TLR) -2, lipopolysaccharide-binding protein (LBP), and CD14, whereas TLR-4 and MD-2 are not involved. J Biol Chem.

[b37] Srivastava A, Henneke P, Visintin A, Morse SC, Martin V, Watkins C (2005). The apoptotic response to pneumolysin is Toll-like receptor 4 dependent and protects against pneumococcal disease. Infect Immun.

[b38] Takeuchi O, Hoshino K, Kawai T, Sanjo H, Takada H, Ogawa T (1999). Differential roles of TLR2 and TLR4 in recognition of gram-negative and gram-positive bacterial cell wall components. Immunity.

[b39] Yoshimura A, Lien E, Ingalls RR, Tuomanen E, Dziarski R, Golenbock D (1999). Cutting edge: recognition of Gram-positive bacterial cell wall components by the innate immune system occurs via Toll-like receptor 2. J Immunol.

